# 腺病毒介导的ING4基因对人肺腺癌裸鼠移植瘤的生长抑制作用及其分子机制

**DOI:** 10.3779/j.issn.1009-3419.2014.02.13

**Published:** 2014-02-20

**Authors:** 锦宏 黄, 吉成 杨, 春华 凌, 大国 赵, 宇锋 谢, 振华 由

**Affiliations:** 1 215500 常熟，常熟市第二人民医院呼吸内科 Department of Respiratory, Second People's Hospital of Changshu, Changshu 215500, China; 2 215123 苏州，苏州大学医学部基础医学与生物科学学院细胞与分子生物学教研室 Department of Cell and Molecular Biology, College of Medicine, Soochow University, Suzhou 215123, China; 3 215006 苏州，苏州大学附属第一医院呼吸内科 Department of Respiratory Medicine, First Affiliated Hospital of Soochow University, Suzhou 215006, China

**Keywords:** 生长抑制因子4, 肺肿瘤, 移植瘤, Inhibitor of growth 4, Lung neoplasms, Xenografts

## Abstract

**背景与目的:**

肿瘤生长抑制因子4（inhibitor of growth 4, *ING4*）基因是一种重要的肿瘤抑制因子，研究发现*ING4*基因对多种肿瘤细胞具有抑癌作用。本研究旨在探讨*ING4*基因对人肺腺癌裸鼠移植瘤的生长抑制作用及其潜在的作用机制。

**方法:**

采用SPC-A1细胞株建立肺腺癌裸鼠移植瘤模型，15只荷瘤裸鼠随机等分为PBS组、腺病毒组（Ad-GFP组）、腺病毒介导的ING4组（Ad-ING4组），上述各组进行局部干预用药，动态测量肿瘤体积，治疗结束后摘取瘤体称重并计算瘤重抑瘤率；脱氧核糖核苷酸末端转移酶介导的缺口末端标记（TUNEL）法检测瘤体内细胞凋亡情况，免疫组织化学SP法检测天冬氨酸特异性半胱氨酸蛋白酶-3（Caspase-3）、环氧化酶-2（COX-2）、Fas与FasL的表达。

**结果:**

Ad-ING4组的肿瘤体积、瘤重均呈现明显下降，与Ad-GFP组抑瘤率（1.31%±0.31%）比较，差异有统计学意义（*P* < 0.05），其抑瘤率为（33.17%±5.24%）；Ad-ING4组的凋亡指数为（69.23%±6.53%），与PBS组（17.04%±1.10%）、Ad-GFP组（18.81%±1.93%）比较，差异有统计学意义（*P* < 0.05）。SP法检测结果显示，Ad-ING4可明显上调Caspase-3、Fas与FasL表达，下调COX-2表达。

**结论:**

ING4具有抑制肺腺癌裸鼠移植瘤的生长抑制作用，该作用机制可能与诱导肿瘤细胞凋亡有关。

## Abstract (en)

**Background and objective:**

The inhibitor of growth 4 (*ING4*) is an important tumor suppressive gene. It has been proven that ING4 could inhibite the proliferation of many tumors. The aim of this study is to investigate the inhibitory effect and anti-cancer mechanism of adenovirus-mediated *ING4* gene on SPC-A1 human lung adenocarcinoma in nude mice.

**Methods:**

A human lung adenocarcinoma xenograft model was established with SPC-A1 cells in nude mice. A total of 15 tumor-bearing nude mice were randomly divided into three groups, namely, PBS, Ad-GFP, and Ad-ING4. The mice in the three groups were intratumorally injected every other day. Their tumor volumes were continually recorded. The treatment tumors were then removed from the mice and weighed. Tumor inhibition rates were calculated. Cell apoptosis was examined by TUNEL method. Caspase-3, COX-2, Fas, and FasL expressions were investigated by immunohistochemistry SP assay.

**Results:**

Both tumor weight and volume in the Ad-ING4 group were significantly decreased. The tumor inhibition rate of the mice in the Ad-ING4 group (33.17%±5.24%) was statistically different from that of the mice in the Ad-GFP group (1.31%±0.31%; *P* < 0.05). The apoptotic index of the mice in the Ad-ING4 group (69.23%±6.53%) was also significantly different from those in PBS (17.04%±1.10%) and Ad-GFP groups (18.81%±1.93%; *P* < 0.05). Based on immunohistochemistry SP assay, the results showed that Ad-ING4 may not only upregulate the expressions of caspase-3, Fas, and FasL but also downregulate the expression of COX-2.

**Conclusion:**

*ING4* gene elicited a remarkable growth inhibitory effect on human lung adenocarcinoma xenografts in nude mice. The mechanism is possibly related to an increase in tumor cell apoptosis.

生长抑制因子4（inhibitor of growth 4, *ING4*）基因属生长抑制因子（ING）家族成员，最初在人脑垂体中被分离出来^[1]^。该基因定位于染色体12p13-31区域内，由8个外显子和7个内含子组成，cDNA全长1, 380 bp，编码蛋白含249个氨基酸分子^[2]^。生物信息学分析显示ING4有一个PHD（plant homeodomain）锌指区和核定位信号（NLS）区。*ING4*基因在多种肿瘤中易发生缺失突变和/或表达下调，并与肿瘤发生和肿瘤恶性程度密切相关^[3, 4]^。外源性*ING4*基因可通过多种途径发挥抗肿瘤效应，对多种肿瘤细胞具有抑癌作用，其抑癌作用与抑制肿瘤细胞生长和肿瘤血管的生成，诱导肿瘤细胞凋亡，调节肿瘤细胞生长周期、引起G_2_/M期阻滞，增强p53活性等有关^[5-8]^；为研究ING4在肺癌基因治疗中的应用，本研究建立SPC-A1细胞肺腺癌裸鼠移植瘤模型，通过腺病毒介导的*ING4*（Ad-ING4）基因治疗，观察ING4对移植瘤的生长抑制作用，并对其可能的分子机制进行了研究，为肺癌的基因治疗提供实验室依据。

## 1 材料与方法

### 1.1 材料

#### 1.1.1 主要试剂

人肺腺癌细胞株SPC-A1细胞、携带绿色荧光蛋白（GFP）的腺病毒（adenovirus Ad-GFP）和腺病毒介导的ING4（Ad-ING4）均由苏州大学基础医学系细胞与分子生物学教研室提供；培养基RPMI-1640，胎牛血清购自Gibcol Brl公司；TUNEL细胞凋亡检测试剂盒购自北京碧云天生物技术研究所；天冬氨酸特异性半胱氨酸蛋白酶-3（Caspase-3）、环氧化酶-2（COX-2）、Fas、FasL一抗、二抗均购自福州迈新生物技术公司（进口分装）。

#### 1.1.2 实验动物

3周-4周龄，体重约20 g，雄性BALB/cnu/nu裸鼠15只，购自中科院上海斯莱克实验动物中心[许可证号：SCXK(沪)2007-0005]，饲养于苏州大学实验动物中心SPF级动物室，自由摄入经过严格灭菌处理的饲料及水，实验操作遵守无菌原则。

### 1.2 方法

#### 1.2.1 细胞的培养

SPC-A1细胞用RPMI-1640完全培养基（10%FCS），在37 oC、5%CO_2_的培养箱内培养，2天-3天传代一次，取对数生长期的细胞进行造模。

#### 1.2.2 肺腺癌裸鼠移植瘤模型的建立

取对数生长期的SPC-A1肺腺癌细胞，用PBS调整细胞浓度制备3.0×10^7^/mL的细胞悬液，于裸鼠右前腋皮下接种细胞悬液100 µL，隔日观察并记录肺癌细胞在裸鼠体内的生长和成瘤情况。

#### 1.2.3 Ad-ING4对裸鼠移植瘤生长的影响

按常规法^[9]^进行，即接种2周左右且肿瘤体积约为0.15 cm^3^时，应用抽签法将15只裸鼠随机均分为3组，即：①PBS组：给50 µL PBS/只；②Ad-GFP组：给Ad-GFP 50 µL（1.5×10^9^ pfu/mL）/只；③Ad-ING4组：给Ad-ING4 50 µL（1.5×10^9^ pfu/mL）/只；各组均采用瘤体内注射干预用药，隔日1次，共注射6次。第1次治疗前及开始治疗后隔日测量各组瘤体的瘤长径（L）和短径（S），根据公式V（cm3）=L ×S^2^×0.5（V：瘤体积，L：长径，S：短径），绘制瘤体体积-时间变化曲线；治疗15 d后，将裸鼠脱颈处死，取瘤体组织称重，计算抑瘤率：抑瘤率（%）=（1-实验组平均瘤重/对照组平均瘤重）×100%。

#### 1.2.4 肿瘤组织病理学检查及凋亡指数测定

将肿瘤组织用10%中性甲醛固定过夜，常规石蜡包埋，切片，常规HE染色，用以观察各组肿瘤组织细胞的形态变化，初步判断SPC-A1肺腺癌细胞的凋亡情况。用脱氧核糖核苷酸末端转移酶介导的缺口末端标记法（TUNEL）检测细胞凋亡，具体操作按试剂盒说明书进行。细胞核呈棕褐色或棕黄色颗粒且具备凋亡细胞形态学特征判定为凋亡细胞。每张切片在高倍视野下（×400）取5个视野，分别计数凋亡细胞数和细胞总数，计算凋亡指数：凋亡指数（apoptotic index, AI）=凋亡细胞数/细胞总数×100%。

#### 1.2.5 免疫组织化学SP法检测凋亡相关因子的表达

取上述各组瘤体组织，用免疫组织化学SP法染色，检测Caspase-3、COX-2、Fas与FasL凋亡相关因子的表达。以高倍镜（×400）下10个视野的阳性细胞数，取平均值（阳性细胞为细胞质内呈弥漫状分布的棕黄色颗粒）作为各凋亡相关因子的表达强度。

### 1.3 统计学处理

用SPSS 16.0统计软件进行数据处理。实验数据用Mean±SD表示，采用*t*检验，以*P* < 0.05为差异有统计学意义。

## 2 结果

### 2.1 Ad-ING4对裸鼠移植瘤生长的影响

本实验中成功地建立了SPC-A1肺腺癌裸鼠移植瘤模型，成瘤率为100%。治疗15 d后，Ad-ING4组的肿瘤体积及重量明显小于PBS组和Ad-GFP组，差异有统计学意义（*P* < 0.05）；Ad-ING4组的抑瘤率（33.17%±5.24%）与Ad-GFP组（1.31%±0.31%）比较，差异有统计学意义（*P* < 0.05）（图 1）。

**1:**
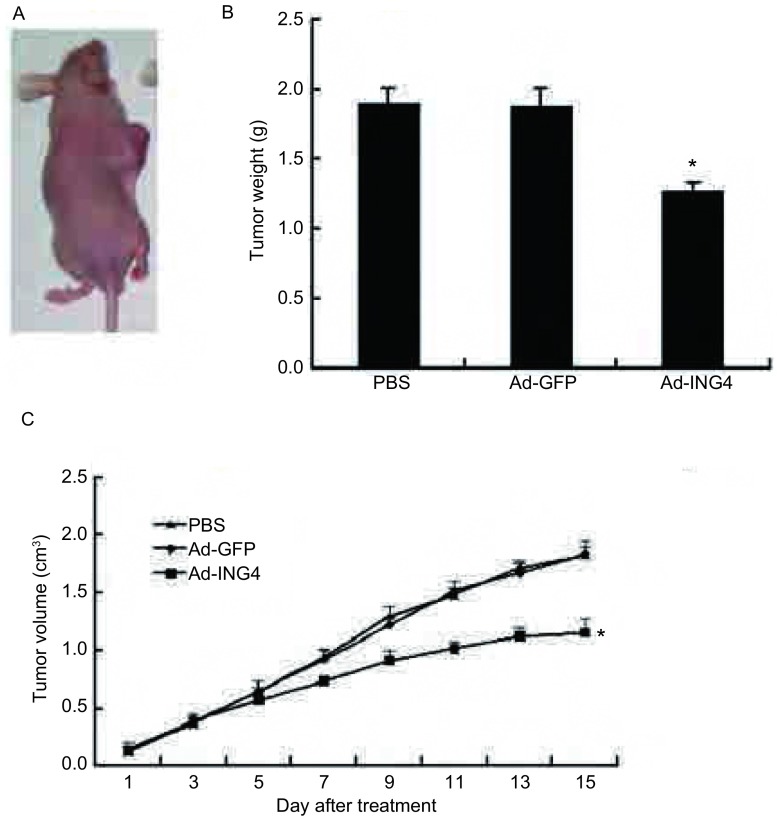
肺腺癌裸鼠移植瘤生长抑制比较（与PBS组、Ad-GFP组比较，*P < 0.05）。A：动物实验大体图，可见种植后有瘤体生长；B：瘤体重量比较；C：各组瘤体体积-时间变化曲线 Inhibition of human lung adenocarcinoma xenografts by Ad-ING4 (*P < 0.05 compared with PBS and Ad-GFP group). A: The pictures of human lung adenocarcinoma xenografts; B: The weight change of human lung adenocarcinoma xenografts treated with Ad-ING4; C: The curves of tumor volume of human lung adenocarcinoma xenografts after treatment

### 2.2 瘤体组织病理学检查

各组瘤体组织进行HE染色，高倍镜下观察各组肿瘤组织细胞凋亡情况，肿瘤细胞凋亡的判断标准：细胞体积变小，细胞质浓缩，细胞核固缩、碎裂、溶解，组织间有大量空泡形成。结果显示：PBS组和Ad-GFP组瘤体组织的肿瘤细胞排列密集，癌细胞异型性明显，未出现上述细胞凋亡特征；Ad-ING4组中大量细胞呈细胞核固缩、裂解或溶解，细胞质浓缩，细胞膜不完整，组织间有大量空泡形成，呈现明显的细胞凋亡形态特征（图 2）。

**2:**
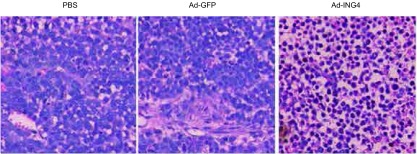
肺腺癌裸鼠移植瘤病理学观察结果（HE，× 400）。PBS组和Ad-GFP组仅见少许坏死，大部分为肿瘤细胞；Ad-ING4组坏死细胞较多，肿瘤细胞较少 The pictures of cellular morphologic of human lung adenocarcinoma xenografts (HE, ×400). PBS group and Ad-GFP group: a few necrosis cells, tumor cells grow well in nude mice with transfection; Ad-ING4 group: a lot of necrosis cells are presented in the tumor tissues in nude mice with transfection, while a few tumor cells

### 2.3 TUNEL检测细胞凋亡

结果显示，细胞核中有棕黄色着染者为阳性细胞，染色质凝聚、浓缩，呈凋亡细胞的形态学征象，部分阳性细胞核染色质仍很疏松，部分呈圆形深染的棕黄色小体，为典型的凋亡小体。各组凋亡的细胞数目存在差别，Ad-ING4组的凋亡指数（69.23%±6.53%），与PBS组（17.04%±1.10%）、Ad-GFP组（18.81%±1.93%）比较，差异有统计学意义（*P* < 0.05）。

### 2.4 瘤体组织中凋亡相关因子的表达

免疫组织化学SP法染色结果显示，Ad-ING4组的Caspase-3、Fas与FasL阳性细胞数均明显高于PBS组和Ad-GFP组，差异有统计学意义（*P* < 0.05）；Ad-ING4组的COX-2阳性细胞数低于PBS组和Ad-GFP组，差异有统计学意义（*P* < 0.05）（表 1，图 3）。

**1:** 各组肿瘤组织中相关因子表达的比较 Expression change of cytokines in human lung adenocarcinoma xenografts

Group	Caspase-3	COX-2	Fas	FasL
PBS	13.42±3.69	112.64±11.21	13.53±6.09	14.01±3.87
Ad-GFP	15.39±3.04	109.72±9.57	14.76±3.66	15.78±4.81
Ad-ING4	43.13±6.07^*^	72.33±7.10^*^	32.98±4.49^*^	39.35±5.23^*^
^*^P < 0.05 compared with PBS and Ad-GFP group.				

**3:**
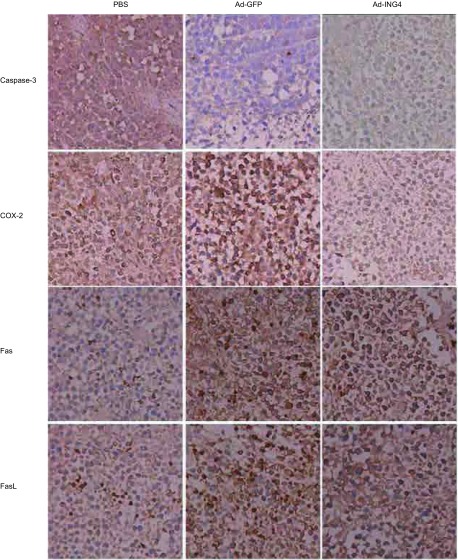
免疫组化染色显示Caspase-3、COX-2、Fas和FasL的表达（SP，× 400） Expression change of Caspase-3, COX-2, Fas and FasL(SP, × 400)

## 3 讨论

基因治疗是将人类的正常基因或有治疗作用的外源目的基因，通过一定的基因转运系统导入人体靶细胞或直接注入人体，表达具有功能的蛋白质，以达到补充、纠正基因的缺陷，从而达到治疗疾病的目的^[10]^。而肺癌是目前世界上发病率最高的恶性肿瘤，也是癌症死亡的主要病因之一^[11]^。因此，针对肺癌的基因治疗成为当今研究的热点之一。

本研究采用SPC-A1细胞株建立了肺腺癌裸鼠移植瘤模型，并进行*ING4*基因治疗，治疗结果显示与对照组（PBS组、Ad-GFP组）比较，Ad-ING4组的肿瘤体积、瘤重明显减小，抑瘤率明显增加，TUNEL检测显示Ad-ING4组移植瘤的凋亡指数也增加，说明*ING4*基因对肺癌可能通过诱导肿瘤细胞凋亡来抑制肿瘤生长。

细胞凋亡包括两条主要途径，即外源性途径和内源性途径，而细胞凋亡最终都需通过天冬氨酸特异性半胱氨酸蛋白酶（Caspase）级联反应来实现，其中Caspase-3处于核心位置，是凋亡级联反应中的关键蛋白酶^[12, 13]^。张等^[6]^研究认为ING4能诱导T24膀胱癌细胞中的*Bax*基因转录明显上调，*Bcl-2*基因的转录水平明显下降，使Bcl-2/Bax的比值下降，最终促使Caspase-3被激活，从而发挥促凋亡作用。Fas是一种重要的死亡受体，属于肿瘤坏死因子受体家族（tumor necrosisfactor receptor, TNFR），是细胞凋亡的主要受体分子。FasL为Fas的天然配体，当Fas与FasL结合后可依次激活Caspase-3、Caspase-6、Caspase-7诱导细胞凋亡^[14]^；本研究通过免疫组化显示，Ad-ING4能使肺癌移植瘤细胞中Fas/FasL、Caspase-3阳性细胞数均明显增加，表明Fas/FasL凋亡因子上调，可使Caspase-3被进一步激活，从而加速细胞凋亡。

COX-2是前列腺腺素生成过程中的限速酶，COX-2在包括肺癌在内的多种肿瘤组织中均呈高表达，它可通过合成致癌物质，从而抑制肿瘤细胞凋亡，促进肿瘤血管新生，促进肿瘤侵袭和转移^[15, 16]^。本研究显示，Ad-ING4组中COX-2阳性细胞数明显下降，表明抑制COX-2的高表达，从而调节*Caspase-3*基因表达水平，可能是*ING4*基因促进肺癌细胞凋亡作用的另一分子机制。

综上所述，本研究初步显示，腺病毒介导的*ING4*基因对SPC-A1肺腺癌裸鼠移植瘤具有生长抑制作用，其机制可能与明显上调Caspase-3、Fas/FasL，下调COX-2的表达相关；然而ING4的抑癌作用是否还存在其他的分子机制尚有待进一步研究，从而为*ING4*基因治疗肺癌提供更充分的实验依据。
